# Recent updates on neuropharmacological effects of luteolin

**DOI:** 10.17179/excli2018-1041

**Published:** 2018-02-28

**Authors:** Gaurav Gupta, Juhi Tiwari, Rajiv Dahiya, Rakesh Kumar Sharma, Anurag Mishra, Kamal Dua

**Affiliations:** 1 School of Pharmaceutical Sciences, Jaipur National University, Jagatpura 302017, Jaipur, India; 2 Laboratory of Peptide Research and Development, School of Pharmacy, Faculty of Medical Sciences, The University of the West Indies, St. Augustine, Trinidad & Tobago, West Indies; 3 School of Pharmacy, Suresh Gyan Vihar University, Jagatpura 302017, Jaipur, India; 4 Discipline of Pharmacy, Graduate School of Health, University of Technology Sydney, Sydney, NSW 2007, Australia; 5 School of Pharmaceutical Sciences, Shoolini University, Solan, Himachal Pradesh, 173229, India

## ⁯

Dear Editor, 

Luteolin (3,4,5,7-tetrahydroxyflavone) is a naturally found flavone, which is obtained from numerous plant species (Kim and Kim, 2012[5]). Chemically, it has a C6-C3-C6 structure that contains two benzene rings and one oxygen-containing ring with a C2-C3 carbon double bond. Structure-activity studies (SAS) have revealed that the presence of hydroxyl moieties at carbons 5, 7, 3 and 4 positions of the luteolin structure and the presence of the 2-3 double bond are accountable for its numerous pharmacological activities (Lin et al., 2008[8]). Luteolin is naturally found as a glycosylated form, is existing in several types of fruits and vegetables, such as pepper, thyme, broccoli, and celery (Lopez-Lazaro, 2009[10]). Various research studies have confirmed that luteolin possesses antioxidant, anticancer, anti-inflammatory, and neuroprotective effects; though, a coherent review of the scientific literature related to its neuroprotective effects is still lacking. 

In this letter, conclusive evidences have been presented for the potent antioxidant activity of luteolin reported in various *in vitro *and* in vivo* studies (Table 1(Tab. 1); References in Table 1: Wang et al., 2017[19]; Kim et al., 2017[6]; Zhang et al., 2017[25]; Tambe et al., 2017[16]; Shen et al., 2016[15]; Wang et al., 2016[18]; Zhen et al., 2016[26]; Burton et al., 2016[2]; Yu et al., 2015[24]; Lamy et al., 2015[7]; Fu et al., 2014[4]; Bandaruk et al., 2014[1]; Xu et al., 2014[20]; Patil et al., 2014[12]; Zhu et al., 2014[27]; Yan et al., 2014[22]; Wang et al., 2015[17]; Xu et al., 2014[21]; Nazari et al., 2013[11]; Yoo et al., 2013[23]; Liu et al., 2013[9]; Qiao et al., 2012[13]; Qiao et al., 2012[14]). Luteolin also reduces inflammation in brain tissues and in regulating different cell signaling pathways (Dirscherl et al., 2010[3]). Oxidative stress and neuro-inflammation are possible drivers of neurodegeneration. Thus, a chemical moiety like luteolin with potential antioxidant and anti-inflammatory activity could be used as a therapeutic agent for neurodegenerative diseases. 

## Conflict of interest

The authors declare no conflict of interest.

## Figures and Tables

**Table 1:**
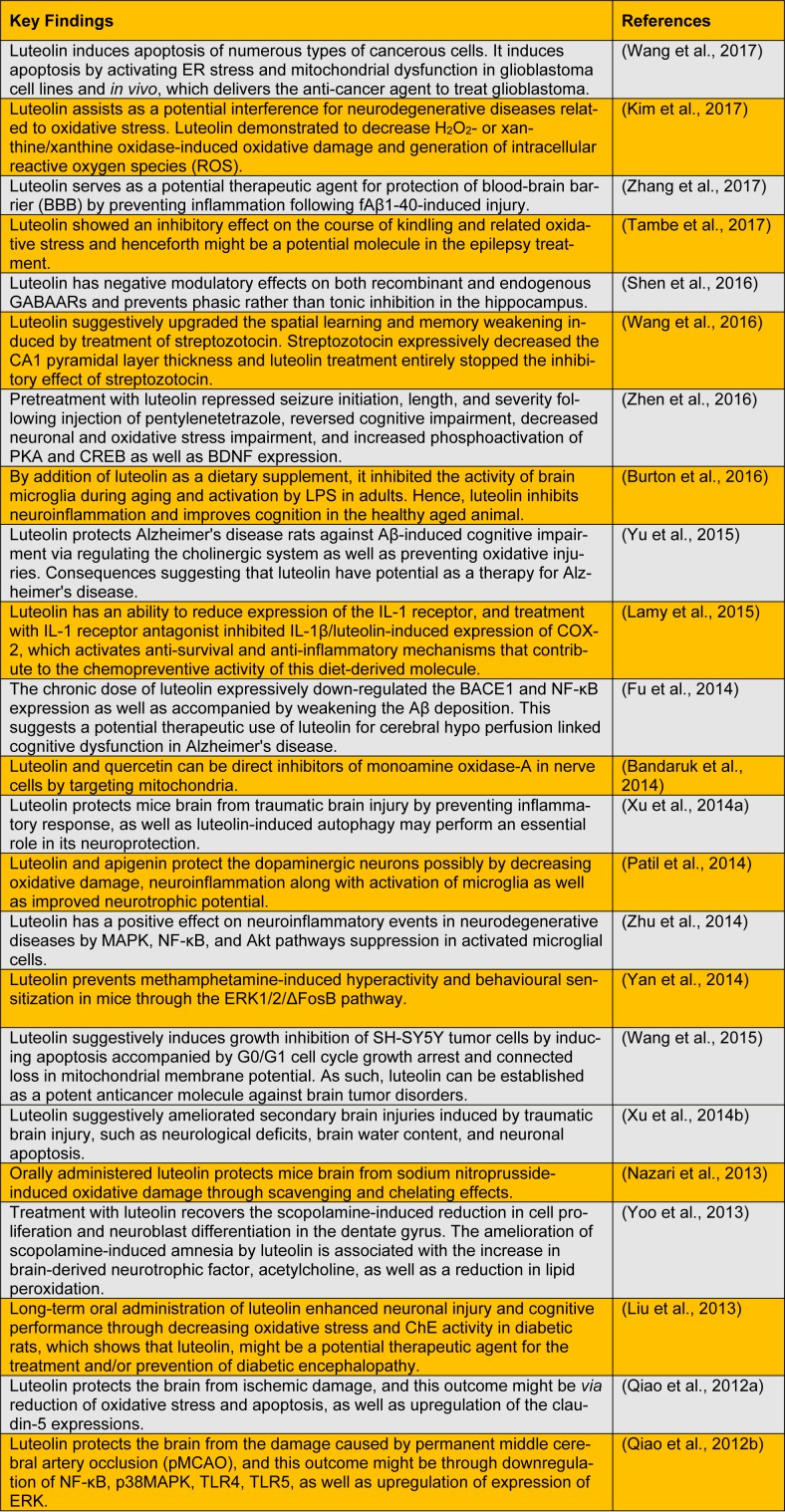
Recent updates on neuropharmacological effects of luteolin
